# Phase II study of preoperative bevacizumab, capecitabine and radiotherapy for resectable locally-advanced rectal cancer

**DOI:** 10.1186/s12885-015-1052-0

**Published:** 2015-02-26

**Authors:** Margarita García, Mercedes Martinez-Villacampa, Cristina Santos, Valentin Navarro, Alex Teule, Ferran Losa, Aleydis Pisa, Maria Cambray, Gemma Soler, Laura Lema, Esther Kreisler, Agnes Figueras, Xavier San Juan, Francesc Viñals, Sebastiano Biondo, Ramon Salazar

**Affiliations:** 1 Clinical Research Unit, Institut Català d’Oncologia, Avinguda Gran Via de l’Hospitalet, 199-203 08907 L’Hospitalet de Llobregat, Barcelona, Spain; 2 Department of Medical Oncology, Institut Català d’Oncologia-IDIBELL, L’Hospitalet, Barcelona, Spain; 3 Department of Medical Oncology, Hospital General de L’Hospitalet, Barcelona, Spain; 4 Department of Radiotherapy, Institut Català d’Oncologia-IDIBELL, L’Hospitalet, Barcelona, Spain; 5 Department of Surgery, Hospital Universitario de Bellvitge –IDIBELL, L’Hospitalet, Barcelona, Spain; 6 Translational Research Laboratory, Institut Català d’Oncologia-IDIBELL, L’Hospitalet, Barcelona, Spain; 7 Department of Pathology, Hospital Universitario de Bellvitge –IDIBELL, L’Hospitalet, Barcelona, Spain

**Keywords:** Locally-advanced, Rectal cancer, Bevacizumab

## Abstract

**Background:**

To evaluate whether the addition of bevacizumab (BVZ) to capecitabine-based chemoradiotherapy in the preoperative treatment of locally advanced rectal cancer (LARC) improves efficacy measured by the pathological complete response (pCR) rate.

**Methods:**

A phase II two-step design was performed. Patients received four cycles of therapy consisting of: BVZ 10 mg/kg in first infusion on day 1 and 5 mg/kg on days 15, 29, 43, capecitabine 1800 mg/m^2^/day 5 days per week during radiotherapy, which consisted of external-beam irradiation (45 Gy in 1.8 Gy dose per session over 5 sessions/week for 5 weeks). Six to eight weeks after completion of all therapies surgery was undergone. To profile the biological behaviour during BVZ treatment we measured molecular biomarkers before treatment, during BVZ monotherapy, and during and after combination therapy. Microvessel density (MVD) was measured after surgery.

**Results:**

Forty-three patients were assessed and 41 were included in the study. Three patients achieved a pathological complete response (3/40: 7.5%) and 27 (67.5%) had a pathological partial response, (overall pathological response rate of 75%). A further 8 patients (20%) had stable disease, giving a disease control rate of 95%. Downstaging occurred in 31 (31/40: 77.5%) of the patients evaluated. This treatment resulted in an actuarial 4-year disease-free and overall survival of 85.4 and 92.7% respectively. BVZ with chemoradiotherapy showed acceptable toxicity. No correlations were observed between biomarker results and efficacy variables.

**Conclusion:**

BVZ with capecitabine and radiotherapy seem safe and active and produce promising survival results in LARC.

**Trial registration:**

ClinicalTrials.gov Identifier NCT00847119. Trial registration date: February 18, 2009.

## Background

Patients with locally advanced rectal cancer (LARC), clinical stages II/III, are at risk of both locoregional and distant failure. Advances in surgery, as total mesorectal excision (TME) have improved local control [1-3].

nd neoadjuvant chemotherapy and radiation therapy have significantly enhanced clinical outcome, in terms of reduction in local recurrence and improvements in sphincter-sparing surgeries [4]. Patients with a pathological complete response (pCR) after preoperative chemo-radiotherapy, have a clear advantage in terms of disease-free survival (DFS) and overall survival (OS) [5,6]. Attempts have been made in order to improve the pCR of chemoradiation, further than using bolus or continuous-infusion of 5-fluorouracil (5-FU) and radiation. Capecitabine has the potential to significantly increase the quality of life of patients during treatment, but data comparing it with 5-FU are still early in development and oxaliplatin has failed to show a benefit in efficacy end points [7,8]. Among the most important challenges that remain in the management of patients with this malignancy is the necessity to investigate new treatments that can improve this rate of responders. In addition, tumor regression after preoperative chemoradiation has been suggested to be associated with smaller, less aggressive disease and with the molecular tumor profile regulating treatment response [5]. Bevacizumab (BVZ) is an antibody against human vascular endothelial growth factor (VEGF). Following the vascular normalization hypothesis [9] although speculative, the advantage conferred by the treatment with bevacizumab plus chemotherapy in the treatment of colon cancer patients could be due to increased tumour cell sensitivity to the action of the chemotherapy or even the better delivery of chemotherapy to tumours. Therefore it is of great interest to study combinations of BVZ and chemoradiation to improve the rate of responders. The first study published with BVZ as preoperative treatment in LARC in combination with radiotherapy by Willett et al. has been supplemented with final results of the same study [10]. In this study, two of the first 5 patients had dose-limiting toxicity, consisting of diarrhoea and colitis during combination therapy. Consequently, dose escalation was stopped and a dose of 5 mg/kg of BVZ was recommended for phase II. The authors discussed the possibility that this toxicity (whose profile seems to be as a result of radiotherapy) was due to the elicitation of protective effect of VEGF on intestinal damage caused by radiation therapy combined with chemotherapy. The interesting thing was that precisely these patients were those who showed two pathological complete responses after surgery. These authors found a marked increase in apoptosis of tumour cells on day +12, and a tendency to increased proliferation was attributed to improved tumour microenvironment.

In line with the Willett group the aim of this study was to explore the effects of a dose of BVZ in monotherapy followed by concurrent treatment with BVZ, capecitabine and radiotherapy. The novelty of this study was the administration of a mono-dose of BVZ 10 mg/kg, followed by a lower dose in the concomitant setting in which the effects of the single dose and the clinical, pathological and biological effects after surgery were studied.

The main objective of this study was to evaluate whether the addition of BVZ to capecitabine-based chemoradiotherapy in the preoperative treatment of LARC improves efficacy measured by the pCR rate. Antiangiogenic markers such as vascular endothelial growth factor A (VEGF-A), vascular endothelial cadherin (VE-cadherin) and microvessel density were evaluated in order to try to correlate the results of expression of these markers with the effect obtained on the tumor and also to assess the prognostic value of these markers on the treatment outcome in relation to other known prognostic markers [11].

## Methods

### Patients

Eligible adult patients were required to have histologically confirmed locally advanced rectal adenocarcinoma located < 15 cm from anal verge. The main inclusion criteria were as follows: written informed consent prior to any study related procedure, male and female aged 18 to 75 years, ECOG performance status 0 or 1, clinical stage of T3, T4 with/without regional lymph node metastases, no metastatic disease, no tumour haemorrhage in the week prior to start of study treatment, external derivation in symptomatic occlusive tumour, no prior cancer treatment, adequate bone marrow, hepatic and renal function and less than 10% weight loss. Patients were excluded if they were not amenable to resection, or presented any other malignancy which has been active or treated within the past 5 years, with the exception of in situ carcinoma of the cervix and non-melanoma skin lesions adequately treated. No prior or concurrent significant medical conditions were admitted such as cerebrovascular disease or myocardial infarction within the past year, uncontrolled hypertension while receiving chronic medication, unstable angina, New York Heart Association class II-IV congestive heart failure, serious cardiac arrhythmia requiring medication, major trauma within the past 28 days, serious non-healing wound, ulcer or bone fracture, evidence of bleeding diathesis or coagulopathy or inability to take oral medication. Evidence of metabolic dysfunction, unknown dihydropyrimidine dehydrogenase deficiency, major surgery in the 4 weeks prior to the start of study treatment, no concurrent chronic, daily treatment with aspirin (>325 mg/day) and more than 10 days since prior use of full-dose oral or parenteral anticoagulants for therapeutic purposes were also exclusion criteria. Local Ethical Committee approved the trial: Comité de Ética de Investigación Clínica del Hospital Universitari de Bellvitge, Edifici Unitat de Recerca, Feixa Llarga, s/n, 08907 L'Hospitalet de Llobregat (Barcelona).

### Treatment plan and dose modifications

Bevacizumab was supplied by Roche (Madrid, Spain) in commercially available formulations. Patients received capecitabine plus bevacizumab with concomitant radiotherapy as follows: BVZ initially was administered at 10 mg/kg over a period of 90 minutes (±15 min). Doses of BVZ were administered at 5 mg/kg on days 15th, 29th and 43rd. No modification of the dose of BVZ was allowed throughout the study, unless the patient's weight varied ≥ 10% over the same, compared to baseline body weight. Capecitabine started after the 2nd infusion BVZ (second cycle) at a dose of 900 mg/m2 administered orally (po) every 12 hours (total daily dose of 1800 mg/m2). The first dose of capecitabine was administered in the evening of day 1 of the second cycle BVZ and continued with a dosing schedule of twice daily for 5 days a week. Patients received radiotherapy in the study consisting of a total dose of 45 Gy in 1.8 Gy dose, per session, over 5 sessions/week for 5 weeks (Energy: Megavoltage photons preferably 6–18 Mv). Surgical resection was scheduled 6–8 weeks after therapy completion. The surgical strategy was at the discretion of the surgeon based on the experience of the working group. After recovery from surgery, patients received adjuvant chemotherapy with regimen selection at the discretion of the treating medical oncologist. Toxicities were evaluated according the National Cancer Institute Common Toxicity Criteria 3.0.

### Study assessments

All patients were initially evaluated with a collection of history, physical examination, chest X-ray, complete blood cell (CBC) count, liver and kidney function tests, pregnancy test if female and ECG. Computed tomography (CT) of the abdomen and pelvis was performed; Colonoscopy with biopsy, endoscopic ultrasound and surgical evaluation were performed in all cases. Carcinoembrionync antigen (CEA) was measured at the baseline and after completion of radiation therapy and after surgery.

Magnetic resonance imaging (MRI) of the pelvis was performed on all patients previous to any treatment. On week 3 after the end of treatment an evaluation visit was performed. A radical resection of the rectal tumor along with an appropriate vascular pedicle and accompanying lymphatic drainage was made. For tumors in the mid and lower rectum total mesorectal excision (TME) was carried out. However, for tumors in the upper rectum (at or above 10 cm from the anal margin) the mesorectum was resected at 5 cm or more distal to the tumor. Tumour staging, pathological examination of the tumour type and complications of surgery were evaluated at the post-surgery visit.

The pathological response criteria and were defined as follows: pathologic complete response: the absence of tumor cells in the surgical specimen including lymph nodes (ypT0N0); partial response: the dominant presence of fibrosis and tumor regression greater than 50%; stable disease: the presence of fibrosis with tumor regression less than 50%; progression of disease: absence of tumor response and/or distant metastasis clinically undetected before surgery. Antiangiogenic profile, consisting of plasma samples, was obtained at baseline and weeks 1 and 5, measuring VEGF-A and VE-cadherin. These markers were evaluated by non-invasive techniques enzyme-linked immunosorbent assay (ELISA) and Real-Time PCR. Tumor samples were assessed for MVD by immunohistochemistry at baseline and after surgery.

A radical resection (R0) was defined as the removal of all macroscopic tumor tissue, no evidence of distant metastases, the absence of microscopic tumor tissue, free resection margins and lymphadenectomy extended beyond involved nodes at postoperative pathological examination. A resection was evaluated as non-radical when microscopic (R1 ≤ 1 mm) or macroscopic (R2) residual tumor was found. Tumor downstaging was determined by comparing the pathological stage with the baseline clinical TNM stage.

Adjuvant chemotherapy was administered according to our local protocol as follows: Patients with ypT4 or ypN+ should receive 4 months of adjuvant chemotherapy with oxaliplatin plus fluoropyrimidines (CAPOX –Capecitabine plus Oxaliplatin- for 6 cycles or FOLFOX4 –folinic acid, Fluorouracile and Oxaliplatin- for 8 cycles) and patients with ypT0-3 N0 should receive 6 cycles with capecitabine alone (1250 mg/m^2^/12 h for 14 days every 21 days).

### Study design

The study was an open-label, unicentric, un-controlled phase II study conducted using a Simon optimal two-stage phase II design [12]. The primary efficacy end-point was pCR rate, defined as a complete regression of the tumor with lack of tumoral cells, only fibrosis or mucin after an exhaustive sampling of the tumoral zone. With an α error of 0.05 and β error of 0.20, an initial sample of 18 patients was treated following these rules: If no pathological responses were seen in these first 18 patients, the combination would be rejected for further study; if at least one response was seen, 25 additional patients would enter the study in order to achieve a given standard error of the estimated pCR rate.

Secondary end-points were: overall clinical response rate: percentage of patients with complete and partial clinical response; downstaging rate: percentage of patients with improved tumour staging compared to baseline; local control rate: percentage of patients who get resection R0.

Overall survival (OS), defined as the elapsed time from baseline to date of the death of the patient due to any cause. Disease Free Survival (DFS) determined as time from surgery to recurrence (local or remote) of disease or death from any cause (whichever comes first). Overall survival and DFS were calculated using Kaplan Meier Method. Confidence intervals (CI) at 95% were estimated using normal approximation.

Summary tables (absolute and relative frequencies) were used for the descriptive analysis of categorical variables. Statistical analyses were performed using SAS version 9.2.

Numerical descriptive analyses were performed for VEGF and VE-CAD plasma levels at baseline, week 1 and week 5. The U de Mann–Whitney test was applied in order to detect time changes between baseline vs week 1, baseline vs week 5 and week 1 vs week 5.

Two observers assessed five times microvessel density at each surgery tumor sample, and the value considered for the statistical analysis was the weighted average of all of them. Numerical descriptive analysis was performed for this variable. An univariated Cox regression model was further used in order to evaluate the influence of microvessel density in time efficacy variables.

Association with other prognostic factors was also analysed through Cox proportional hazard model. Data were reported as Hazard Rate Ratios and 95% CI through likehood ratio test. Remark guidelines were followed [13].

## Results

**Figure 1:**
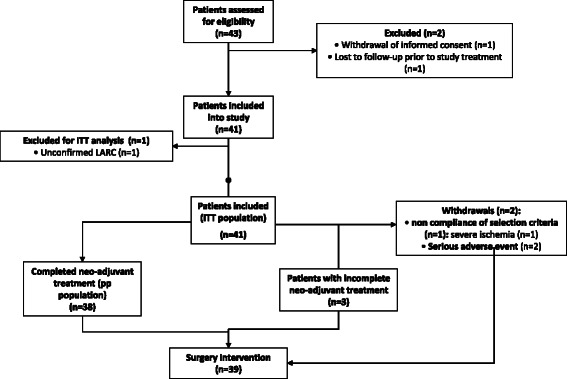
**Disposition of enrolled patients.**

Between July 2007 and July 2010, 43 patients were entered into the study, of whom 2 patients were excluded from the intention to treat (ITT) population because of withdrawal of informed consent (n = 1) and lost to follow-up before the start of treatment protocol (n = 1). One further patient was withdrawn from the analysis because of unconfirmed LARC (Figure 1).

Patient characteristics at baseline are shown in Table 1. Thirty-eight patients completed neo-adjuvant treatment and one patient withdrawn from treatment underwent surgery, therefore in total, 39 patients were surgically treated. Surgery details were as follows: radical surgery was performed in 38 patients (97,4%) and one patient was underwent palliative surgical treatment. Thirty patients were operated by anterior resection (76,9%) and 9 by abdominoperineal amputation (23,1%). Total mesorectal excision was done for 25 patients (69,4%) the remaining patients undergoing surgery had a partial mesorectal excision (PME) due to their tumors were located at the upper rectum, R0 resection was obtained for 33 patients (84,6%) and 31 (79,5%) preserved sphincter function. Hospital length of tay was 14,4 days, mean ± SD, 11.5. Thirteen patients suffered post-operative complications (13/39: 33.3%). Most frequent complications were: wound infection (6: 40%), intraabdominal collection (3: 20%), suture dehiscence (2: 13.3%), ileus paralytic (2: 13.3%) and pelvic/presacral collection (2: 13.3%).

**Table 1:** **Patient characteristics at baseline (n = 40)**

Patient characteristics	Value
Median (range) age, years	63 (54–66)
Gender, n (%)	
Male	30 (75.0)
Female	10 (25.0)
ECOG PS, n (%)	
0	20 (50.0
1	20 (50.0)
TNM status, n (%)	
T3*	32 (80.0)
T3a	3 (7.5)
T3b	1 (2.5)
T3c	2 (5.0)
T4	2 (5.0)
Nx	1 (2.5)
N0	1 (2.5)
N1	24 (60.0)
N2	14 (35.0)

*It was not specified if the T3 status was T3a, T3b or T3c.

Regarding adjuvant treatment: 33 patients received 4 months of adjuvant chemotherapy, 17 patients with capecitabine because they were ypT0-3 N0 and 16 patients received capecitabine or 5FU plus oxaliplatin because they were ypN+ or ypT4. One patient received palliative chemotherapy. Three patients couldn’t receive adjuvant chemotherapy because they had suffered some surgery complications that affected their *performance status*. Finally, 2 patients did not receive chemotherapy because they presented a cardiac problem (one of them at post operative period). Chemotherapy was started 4 to 8 weeks after surgery.

### Efficacy

Three patients had a pathological complete response (3/40: 7.5%) and 27 (67.5%) had a pathological partial response, for an overall pathological response rate of 75%. A further 8 patients (20%) had stable disease, giving a disease control rate of 95%. One of the remaining patients suffered disease progression and another one did not undergo surgery. Of the partial response patients, 8 had a partial microscopical residual response and 18 had a partial macroscopical residual response. One patient did not have this data (Table 2).

**Table 2:** Response of primary tumor and nodal status*

Response	N (%)
ypT:	
0	3 (7.5)
1	1 (2.5)
2	12 (30.0)
3	22 (55.0)
4	1 (2.5)
Not available*	1 (2.5)
ypN:	
0	25 (62.5)
1	13 (32.5)
2	1 (2.5)
Not available*	1 (2.5)
Tumor regression grade:	
1	3 (7.0)
2	8 (20.0)
3	19 (47.5)
4	9 (22.5)
Not available*	1 (2.5)

*One patient did not undergo surgery.

Downstaging occurred in 31 (31/40: 77.5%) of the patients evaluated.

### Safety

In total, 3 patients (7.3%) withdrew from the study. Reasons for study discontinuation were: toxicity or other adverse event or concomitant disease (2, 4.9%) and noncompliance of selection criteria (1, 2.4%).

Leucopenia was present in 5 patients, two of them grade 2 and three grade 3, and neutropenia appeared as grade 1–3 in 7.3% of patients. Lymphopenia was the most commonly observed event, occurring in 9.8% of patients (all related to capecitabine and 4.9% to bevacizumab). Non-hematological adverse events are summarized in Table 3.

**Table 3:** **Nonhematologic Toxicity**

	Grade (no. of patients)			
Toxicity	1	2	3	4
Asthenia	37	2	0	0
Dhiarrea	26	5	1	0
Hypertension	4	1	0	0
Anorexia	9	0	0	0
Disuria	7	0	0	0
Mucositis	8	0	0	0
Abdominal pain	1	6	0	0
Hand-foot syndrome	11	0	0	0
Vasospastic angina	0	0	0	1
Nauseas	4	0	0	0
Radiodermitis	3	1	1	0
Disphonia	3	0	0	0
Rectitis	2	1	0	0
Stomatitis	2	0	0	0
Vomiting	2	0	0	0
Rectorraghia	1	2	0	0
Anal pruritus	3	0	0	0
Other*	1	0	0	0

Worst grade per patient (n = 41).

*Arthralgia, disphonia, eritema, gastric pain, abdominal discomfort, rash, fever, vomiting, tenesmus (1 each one, grade 1–2).

Two patients died during the follow-up period because of pneumonia (n = 1) and pneumothorax (n = 1) that were considered not related to study treatment. At the time of this analysis, only 2 patients have relapsed locally and another 4 patients have presented metastatic disease. Only one patient has died due to underlying cancer.

### Biomarkers profile

Plasma samples were available for 33 patients (details are shown in Table 4). No statistically significant differences were found between baseline levels and levels achieved at week one and 5 (Baseline vs week 1: VEGF 57 pg/ml (*p*-value 0.1763) and VE-cadherin 22 ng/ml (*p*-value 0.3652); Baseline vs week 5: VEGF 16 pg/ml (*p*-value 0.4961) and VE-cadherin 13 ng/ml (*p*-value 0,5469); week 1 vs week 5: VEGF 23 pg/ml (*p*-value 0.6953) and VE-cadherin 10 ng/ml (*p*-value 0.0839) and no correlations were observed between values and efficacy variables. Tumor samples were available for 37 patients. Median MVD was 14 (8,2-33,6) and no correlation was seen with standard prognostic variables as CEA, nodal status, local control, DFS and OS.

**Table 4:** **Descriptive values of angiogenic biomarkers**

		N	NA	Min	Q1	Median	Mean	Q3	Max	Stdev	Var
VEGF (pg/ml)	Baseline	33	12	16,4	57,8	90,5	91,3	114,7	211,2	54,0	2915,9
	Week 1	33	15	3,3	12,0	40,5	47,7	69,3	139,8	41,0	1680,6
	Week 5	33	21	45,6	58,2	79,4	81,4	95,7	158,8	31,8	1014,4
VE-cadherin (ng/ml)	Baseline	33	9	1,5	3,4	4,3	4,4	5,3	9,4	1,8	3,1
	Week 1	33	17	2,5	3,5	3,9	4,5	5,4	7,3	1,4	2,1
	Week 5	33	22	3,1	3,8	5,0	5,0	5,7	7,3	1,4	1,9

N: number; NA: not available; Min: minimum; Q1; quartile 1; Q3: quartile 3; Max: maximum; STdev: standard deviation; Var: variance.

### Outcome

The median follow up of the study population is 40 months, range 13 to 57, local control rate at 4 years is 89.7% (95% CI 67–97), median not reached and DFS rate at 4 years is 68.7% (95% CI 51–81) median not reached with an OS rate of 91.8% at 4 years (95% CI 80–96.7), median not reached.

## Discussion

This is a study with BVZ combined with chemoradiotherapy in patients with LARC that was designed to achieve a 25% of pCR rate. Although the main endpoint was not reached, higher than expected rates of partial microscopical residual response, R0 resection, sphincter-preservation and tumor downstaging were observed, and moreover, data of DFS and OS are encouraging.

Several phase I and II trials have tested the combination of BVZ administered concomitantly with chemo-radiotherapy in patients with LARC in different schedules, in order to assess their efficacy and safety and to study their biological behavior. All of these studies had the expectation of improving the pathological complete response rate to therapy as a surrogate marker of the improvement in disease and overall survival [10,14-24].

Table 5 shows a summary of studies containing bevacizumab which have been published so far. The first point which can be drawn from these studies is that most of them have demonstrated the feasibility of the administration of the combination of these four therapeutic strategies (chemotherapy, radiotherapy, and surgery and antiangiogenic therapy). With the exception of studies of Dipetrillo et al. and Resch et al. [22] all conclude that this combination of treatments can be administered in a realistic and affordable approach to the population of patients with this disease. Similarly, the trial of our group shows a treatment schedule feasible and tolerable. The adverse event profile observed during this study was comparable to those reported in other studies involving capecitabine plus bevacizumab with concurrent radiotherapy.

**Table 5:** **summary of studies with chemoradiotherapy plus bevacizumab**

Study	Phase	N. of patients	Staging	Radiotherapy Gy/fractions/weeks	Bevacizumab	Concomitant chemotherapy	pCR N.%	Downstaging N.%	R0%
Czito et al. [14]	I	11	II-IV	50.4/28/5.5	15 mg/Kg 1st dose	Capecitabine: 500–825 mg/m^2^/bid 5 days/week	2/11 18.2%	Overall: 81.8%	81.8%
					10 mg/Kg days 8, 22	Oxaliplatin 50–75 mg/m^2^/1, 8, 15, 22, 29			
Willett et a [10]	II	32	T3-T4	50.4/28/5.5	5-10 mg/Kg days 1, 8, 15, 22	Fluorouracile: 225 mg/m^2^/24 hours/4 cycles	5/32 16%	T stage: 50% N stage: 56.5%	93.7%
Crane et al. [16]	II	25	T3N0/T3N1	50.4/28/5.5	5 mg/Kg 3 doses	Capecitabine: 900 mg/m^2^/bid 5 days/week	8/25 32%	T stage: 64% N stage: 15%	100%
Nogué et al. [18]	II	47	T3N0-T4N2	50.4/25/5.5	7 mg/Kg induction 4 cycles	Capecitabine: 825 mg/m^2^/bid 5 days/week	16/45 36%	-	97.9%
					5 mg/Kg days 1, 15, 29				
Koukourakis et al. [17]	II	19	T3 and/or N+	Hypofractionated accelerated	5 mg/Kg 2 doses	Capecitabine: 600 mg/m^2^/bid 5 days/week	7/19 36.8%	-	-
[19]	II	61	T2N1-T4N2	50.4/28/5.5	5 mg/Kg days −14, 1, 15, 29	Capecitabine: 825 mg/m^2^/bid 5 days/week	8/60 13.3%	T stage: 64% N stage: 15% Overall: 73.8%	95%
Dipetrillo et al. [20]	II	26	T2N0-T4Nx	50.4/25/5.5	5 mg/Kg days induction and then days 1, 15, 29	Fluorouracile: 200 mg/m^2^/24 hours	5/25 20%	-	-
						Oxaliplatin 50 mg/m^2^/1, 8, 15, 22, 29, 36			
Landry et al. [24]	II	57	T3-T4	50.4/28/5.5	5 mg/Kg days 1, 15, 29	Capecitabine: 825 mg/m^2^/bid 5 days/week	9/57 17%	Overall: 59%	-
						Oxaliplatin 50 mg/m^2^/1, 8, 15, 22, 29			
Gasparini et al. [21]	II	43	T2N1-T4N2	50.4/28/5.5	5 mg/Kg days −14, 1, 15, 29	Capecitabine: 825 mg/m^2^/bid 5 days/week	6/43 14%	T Stage: 34.9% N stage: 41.86%	-
Spigel et al. [23]	II	35 (cohort A)	II/III	50.4/28/5.5	5 mg/Kg days 1, 15	Fluorouracile: 225 mg/m^2^/24 hours days 1 to 42	10/35 29%	-	-
Resch et al. [22]	II	8	cT3	45/-/5	5 mg/Kg days 1, 15, 29	Capecitabine: 825 mg/m^2^/bid 5 days/week 4 weeks	2/8 25%	Overall: 37.5%	-
Kennecke et al. [15]	II	42	T2-T4 N2	50.4/28/5.5	5 mg/Kg days −14, 1, 15, 29	Capecitabine: 825 mg/m^2^/bid days 1–14, 22-35	7/42 18.4%	-	92%
						Oxaliplatin 50 mg/m^2^/1, 8, 22, 29			

Particularly striking is the disparity between the toxicity observed by Dipetrillo et al. and Resch et al. [22] compared to other studies, which a priori does not seem attributable to differences in the doses of the individual components of the treatment regimens. Furthermore, there is some variability with respect to the sample of patients included in each of them, which could produce an effect on results due to selection bias, which consistently operates in the literature against the outcomes observed in phase 2 studies. Similarly, the results in terms of pathologic complete response rates vary from 7.5% in our case, to 36% in the case of Nogué et al. [18], although such discrepancies have been observed previously with other phase 2 trials in these patients. Placed in the context of previous larger phase III studies with chemo-radiotherapy (13.9% to 19.2% pCR rates in Gerard et al. study and 16% in the study of Aschele et al.) [7,8] and pooled analyses (pCR rate of 15.6%) [6] despite the inconsistency in the results, which may be due to interobserver variability or the increasing rate of pCR rate reported after a longer interval between the chemoradiotherapy and surgery without reduction in local relapse and overall survival [25] these rates remain promising.

A long follow-up is needed to assess the impact on other and more important efficacy endpoints, as DFS and OS. In our study, despite the narrow pathological complete response rate, the prolonged median follow-up shows the good results that so far have been observed in survival. If the use of bevacizumab in the treatment of primary rectal tumors improves survival outcomes of patients and disease-free survival, it is something that for now remains hypothetical. Data reported by Willet et al. [26] and the results derived from our study suggest that the antiangiogenic therapy with bevacizumab can prevent the emergence and establishment of metastases in these tumors because of the promising survival rates (69% DFS and 95% OS at 5 years), although further randomized studies are warranted.

Results of this study have justified a randomized phase II trial of this regimen, performed by the Spanish TTD Collaborative Group (AVAXEL trial) whose results are awaited.

## Conclusion

The administration of bevacizumab in addition to a standard neoadjuvant capecitabine-based chemoradiotherapy regimen in patients with LARC is feasible and its efficacy is sufficient to justify further randomized studies. Biological data concurrent to standard treatment can add information to elucidate the role of antiangiogenic treatment. Current long-term follow-up results are promising and can help to determine the benefits of adding bevacizumab to the regimen.

## Abbreviations

5-FU: 5-fluorouracil
BVZ: Bevacizumab
CBC: Complete blood cell
CEA: Carcinoembrionync antigen
CT: Computed tomography
DFS: Disease-free survival
ECG: Electrocardiogram
ELISA: Enzyme-linked immunosorbent assay
ITT: Intention to treat
LARC: Locally advanced rectal cancer
MRI: Magnetic resonance imaging, MVD: microvessel density
OS: Overall survival
pCR: Pathological complete response
po: Administered orally
R0: Radical resection
TME: Total mesorectal excision
VE-CAD: Vascular endothelial cadherin
VEGF: Vascular endothelial growth factor
